# Molecular characterization of intestinal protozoa in two poor communities in the State of São Paulo, Brazil

**DOI:** 10.1186/s13071-015-0714-8

**Published:** 2015-02-15

**Authors:** Érica Boarato David, Semíramis Guimarães, Ana Paula de Oliveira, Teresa Cristina Goulart de Oliveira-Sequeira, Gabriela Nogueira Bittencourt, Ana Rita Moraes Nardi, Paulo Eduardo Martins Ribolla, Regina Maura Bueno Franco, Nilson Branco, Fabio Tosini, Antonino Bella, Edoardo Pozio, Simone M Cacciò

**Affiliations:** Parasitology Department, Institute of Bioscience, São Paulo State University (UNESP), Campus de Botucatu, Botucatu, São Paulo PO Box: 510, CEP: 18618-970 Brazil; Department of Animal Biology, Biology Institute, State University of Campinas, Rua Monteiro Lobato 255, Campinas, São Paulo PO Box: 6109, CEP: 13083-970 Brazil; Department of Infectious, Parasitic and Immunomediated Diseases, IstitutoSuperiore di Sanità, Viale Regina Elena, 299, Rome, 00161 Italy

**Keywords:** Brazil, Intestinal protozoa, Humans, Dogs, River water, Molecular typing

## Abstract

**Background:**

Several species of protozoa cause acute or chronic gastroenteritis in humans, worldwide. The burden of disease is particularly high among children living in developing areas of the world, where transmission is favored by lower hygienic standards and scarce availability of safe water. However, asymptomatic infection and polyparasitism are also commonly observed in poor settings. Here, we investigated the prevalence of intestinal protozoa in two small fishing villages, Porto Said (PS) and Santa Maria da Serra (SM), situated along the river Tietê in the State of São Paolo, Brazil. The villages lack basic public infrastructure and services, such as roads, public water supply, electricity and public health services.

**Methods:**

Multiple fecal samples were collected from 88 individuals in PS and from 38 individuals in SM, who were asymptomatic at the time of sampling and had no recent history of diarrheal disease. To gain insights into potential transmission routes, 49 dog fecal samples (38 from PS and 11 from SM) and 28 river water samples were also collected. All samples were tested by microscopy and PCR was used to genotype *Giardia duodenalis*, *Blastocystis* sp., *Dientamoeba fragilis* and *Cryptosporidium* spp.

**Results:**

By molecular methods, the most common human parasite was *Blastocystis* sp. (prevalence, 45% in PS and 71% in SM), followed by *D. fragilis* (13.6% in PS, and 18.4% in SM) and *G. duodenalis* (18.2% in PS and 7.9% in SM); *Cryptosporidium* spp. were not detected. Sequence analysis revealed large genetic variation among *Blastocystis* samples, with subtypes (STs) 1 and 3 being predominant, and with the notable absence of ST4. Among *G. duodenalis* samples, assemblages A and B were detected in humans, whereas assemblages A, C and D were found in dogs. Finally, all *D. fragilis* samples from humans were genotype 1. A single dog was found infected with *Cryptosporidium canis*. River water samples were negative for the investigated parasites.

**Conclusions:**

This study showed a high carriage of intestinal parasites in asymptomatic individuals from two poor Brazilian villages, and highlighted a large genetic variability of *Blastocystis* spp. and *G. duodenalis*.

## Background

Human infection with gastrointestinal parasites is common worldwide, but a significantly higher morbidity and mortality is observed in developing countries, particularly among children, where many communities still lack access to improved sanitation facilities [1]. Infection can cause acute or chronic gastroenteritis, but a considerable percentage of infected individuals show no symptoms. Among protozoa, species of *Cryptosporidium*, *Blastocystis* and *Entamoeba*, as well as *Giardia duodenalis* and *Dientamoeba fragilis* are the most common etiological causes of intestinal infections.

In many laboratories of developing countries, microscopic examination of stools for detection of cysts, oocysts and trophozoites remains the diagnostic method of choice, due to the low cost of reagents. However, optical microscopy requires considerable technical expertise, is time consuming and, in most cases, does not allow determination of the parasite at the species/genotype level. Molecular methods are more expensive and require specific equipments, but have higher specificity and sensitivity compared to microscopy, and are therefore increasingly used to detect and characterize gastrointestinal protozoa. Furthermore, the large genetic variability observed among isolates of intestinal protozoan species has lead to the description of many genotypes and subtypes that have been found to differ in host range, zoonotic potential and clinical significance [2-4] therefore strongly impacting on our knowledge of the epidemiology of parasitic infection.

Previous surveys on gastrointestinal parasites in humans from Brazil have mainly focused on *Giardia* and *Cryptosporidium*, and investigated the prevalence of infection in risk groups, like children attending daycare centers [5,6], residents of low-income communities [7,8] and HIV patients [9]. Epidemiologic studies have shown that adverse health effects can occur in children infected with multiple gastrointestinal parasites, particularly those from families with extremely low socioeconomic status [5]. However, asymptomatic carriage of single or multiple gastrointestinal parasites has also been reported in many studies [10].

Given the public health significance of the intestinal protozoa infections, the present study was aimed at determining the prevalence and the genetic diversity of intestinal protozoa in humans living in two poor villages situated along the river Tietê in the State of São Paulo, Brazil, and at exploring potential transmission routes by testing animal (dogs) and environmental (river water) samples.

## Methods

### Study area and population

**Figure 1:**
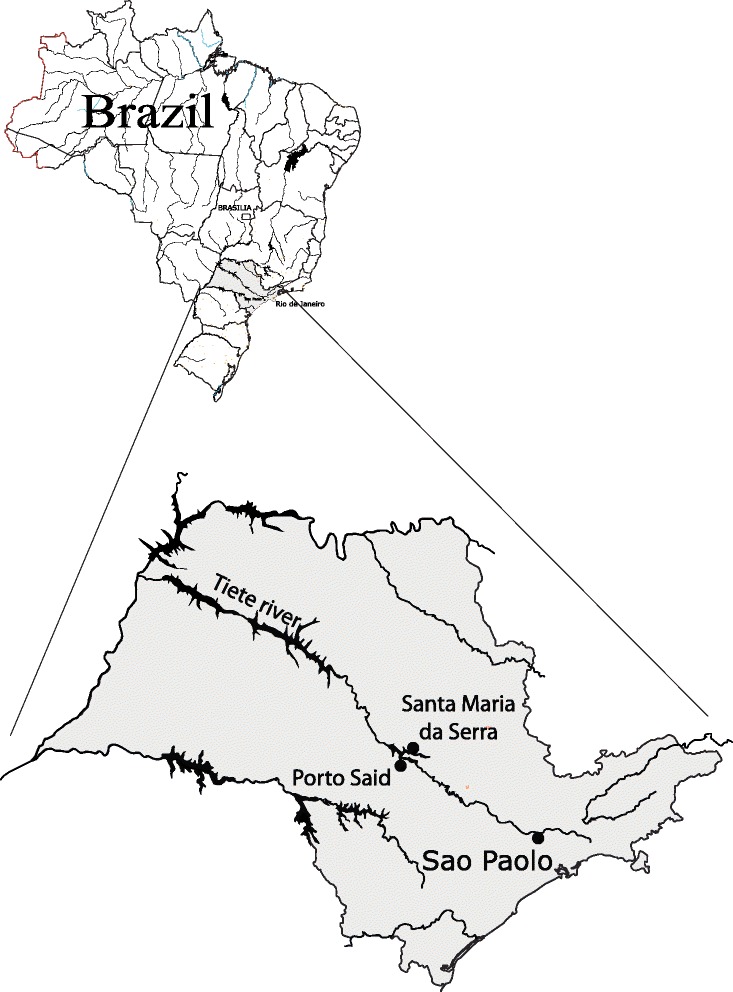
**Map of the area showing the location of the two villages along the Tietê river, State of São Paulo, Brazil.**

This work was conducted in two small fishing villages in the State of São Paulo, Brazil, namely Porto Said (PS), located close to the city of Botucatu (latitude 22°53′9″S, longitude 48°26′42″W), and Santa Maria (SM), located close to the city of Santa Maria da Serra (latitude 22°34′1″S, longitude 48°9′39″W) (Figure 1). These villages are important centers of fishing in the Middle Tietê Basin, and together include approximately 50 families, mostly coming from the poorest regions of the country. These communities lack basic public infrastructure, such as roads, water supply and electricity, and health services. The living conditions are poor, as most families live in makeshift huts covered with plastic and wood, or in small brick and wood houses near the river. For sanitation, homes have cesspools. Drinking water is obtained from cistern wells or from clandestine connections to official networks. Free-roaming animals, including dogs, cats and chickens, are present in both villages. The river is not used as a source of drinking water, but the population is exposed to river water due to occupational or recreational activities.

### Ethical statement

The study was approved by the Research Ethics Committees of the Botucatu Medical School, UNESP (protocol number 3898/2011 CEPE) and Animal Experimentation/Biosciences Institute/UNESP (protocol number 306-CEEA). Individuals provided a written signed informed consent prior to sample collection. The same consent was taken from parents or guardians on behalf of their children.

### Collection and processing of fecal samples

Fecal samples were collected from October 2011 to April 2013. Informed consent was obtained from 88 of the 107 individuals living in the Porto Said village, and from 38 of the 43 individuals living in the Santa Maria village. All individuals received a package with three collectors containing a 2.5% potassium dichromate solution, and were asked to collect three fecal specimens on alternate days. Single fecal samples from dogs (38 in PS and 11 in SM) were also collected in plastic containers and preserved as described above. Fecal samples from chickens and cats could not be collected because of lack of consent from bird owners, and due to the risk associated with sampling of cats that were not vaccinated against rabies.

The fecal material (from humans or dogs) was transferred to test tubes and washed three times with distilled water (800xg for three minutes) to remove potassium dichromate.An aliquot of fecal sediment was stained with Lugol’s iodine, and observed at 40X under an optical microscope (Leica DM750). A second aliquot was processed by flotation with zinc sulfate, and examined microscopically by the same procedure.

### River water samples collection and processing

Water samples from the Tietê River were collected during both the dry and the rainy seasons, from four distinct points in PS and from three distinct points in SM. At each site, two samples of five liters each were collected one meter away from the river bank and 10–15 cm below the surface. The turbidity and the pH were measured for each sample. The procedures were performed according to the USEPA 1623 Method [11]. Samples were first filtered through membranes of mixed esters of cellulose (47 mm diameter, nominal porosity of 3 μm, Millipore®), with modifications as described in [12]. After this step, the membranes were scraped with a smooth-edged plastic loop and rinsed with a solution containing 0.1% Tween 80 for 20 minutes. The eluted material was centrifuged for 15 minutes at 1050xg and the sediment was washed once with Milli-Q water and centrifuged again. Immunomagnetic separation (IMS) of *Giardia* cysts and *Cryptosporidium* oocysts was performed using the Dynabeads® GC-combo kit (Idexx Laboratories, USA) according to the manufacturer’s instructions. After IMS, samples were examined by a direct immunofluorescence assay using the Merifluor kit (Meridian Bioscience, Cincinnati, Ohio, USA), andnucleic acid were stained with the fluorogenic dye DAPI (4′,6-diamino-2-phenylindole).

### Molecular analysis of intestinal protozoa

Genomic DNA was extracted from 200 mg of fecal sediments or from purified water samples using the QIAamp® Stool mini kit (Qiagen, Hilden, Germany) following the manufacturer’s instructions. For *Blastocystis* sp., amplification of a ~600 bp fragment of the small subunit ribosomal RNA (ssu rDNA) gene was carried out as described by [13]. For *G. duodenalis*, amplification of a 511 bp fragment of the beta-giardin gene (*bg*), and of a 530 bp of the triose phosphate isomerase (*tpi*) gene followed described protocols [14,15]. For the glutamate dehydrogenase (*gdh*) gene, amplification of a 530 bp fragment [16], or of a 430 bp fragment [17] were performed. For *D. fragilis*, amplification of a ~300 bp fragment of the ssu rDNA gene was performed as previously described [18]. For *Cryptosporidium* spp., a nested PCR protocol for the amplification of a ~590 bp fragment of the ssu rDNA was used [19].

All PCR reactions were performed in a final volume of 50 μl containing 25 μl of 2x GoTaq Green Master Mix (Promega, Madison, WI, USA), 1 μl of each primer (10 pmol/μl), 5 μl of extracted DNA and 18 μl of sterile water. Aliquots (5–10 μl) of PCR reactions were loaded on 1.5% agarose gel stained with ethidium bromide. Positive PCR products were purified using spin columns (QiaQuick PCR purification kit, Qiagen, Hilden, Germany) and sequenced on both strands.

In cases where amplification of more than one microorganism was suggested by the analysis of chromatograms, the corresponding PCR products were cloned using the pGEM®T easy kit (Promega, Madison, WI, USA), following the manufacturer’s instructions. Plasmids were purified using a commercial kit (QiaMiniPrep, Qiagen, Hilden, Germany) and sequenced on both strands using vector primers.

PCR products were sequenced from both strands using the ABI PRISM®BigDye™ Terminator Cycle Sequencing Kit (Applied Biosystems, Life Technologies™, Carlsbad, CA, USA) according to the manufacturer’s instructions. The sequencing reactions were analysed using the ABI PRISM® 3100 automatic sequencer (Applied Biosystems). Chromatograms were edited and assembled using the SeqMan 7.1 software package (DNASTAR, Madison, WI, USA). Species, genotypes or subtypes of parasites were identified by BLAST searches (http://blast.ncbi.nlm.nih.gov/Blast.cgi), and *Blastocystis* alleles were further identified at the *Blastocystis* database (http://pubmlst.org/blastocystis/). Novel sequences from *Blastocystis* sp. have been deposited in the NCBI nucleotide database under accession numbers KP734166 and KP73417.

### Statistical analysis

Data were summarized by absolute frequency and percentage. The differences between proportions were evaluated by the Chi-square test or, when appropriate, Fisher’s exact test. Statistical analyses were carried out using STATA software version 11.2 (Stata Corporation, College Station, TX, USA).

## Results

### Microscopic detection of intestinal parasites

All individuals (n = 126) were considered asymptomatic, since stools were scored as formed at the time of collection and no episode of diarrhea was reported during the two months prior to sample collection. Out of the 126 individuals studied, 83 (65.9%) were positive for at least one intestinal parasite (Table 1). Among the 88 individuals from PS, 24 (27.3%) were positive for *Entamoeba coli*, 6 (6.8%) for *G. duodenalis*, one (1.1%) for *Blastocystis* sp., and one (1.1%) for *Iodamoeba bütschlii*. Among the 38 individuals from SM, 9 (23.7%) were positive for *Blastocystis* sp., 7 (18.4%) for *E. coli*, 6 (15.8%) for *G. duodenalis*, and one (2.6%) for *Endolimax nana*. Concerning helminthes, microscopy revealed three cases of hookworms and one case of *Strongyloides stercoralis* infection in individuals from PS (Table 1). The prevalence of *Cryptosporidium* spp. and *D. fragilis* was not estimated, as diagnosis requires permanent staining of fecal smears.

**Table 1:** **Microscopic prevalence of parasites in human and dog faecal samples from Porto Said and Santa Maria (Tietê river, State of São Paulo, Brazil)**

	**Human samples**	
**Parasite**	**Porto Said (n = 88)**	**Santa Maria (n = 38)**
Hookworms	1	-
*Blastocystis* spp.	-	3
*Entamoeba coli*	20	3
*Giardia duodenalis*	3	2
*Iodamoeba bütschlii*	1	-
*Strongyloides stercoralis*	1	-
*Endolimax nana + Blastocystis*	-	1
*E. coli* + *Blastocystis*	1	2
*G. duodenalis* + *E. coli*	2	1
*G. duodenalis* + Hookworms	1	-
*E. coli* + Hookworms	1	-
*G. duodenalis* + *Blastocystis*	-	2
*G. duodenalis* + *E. coli* + *Blastocystis*	-	1
	**Dog samples**	
**Parasite**	**Porto Said (n = 38)**	**Santa Maria (n = 11)**
*Ancylostoma* spp.	25	3
*Giardia duodenalis*	2	-
*Toxocara canis*	2	-
*Trichuris vulpis*	-	1
*Ancylostoma* spp. + *T. canis*	1	1
*Ancylostoma* spp. + *G. duodenalis*	1	1
*Ancylostoma* spp. + *Spirocerca* spp.	-	1
*Ancylostoma* spp. + *T. vulpis*	-	1

*Giardia duodenalis* cysts were identified in 3 of the 38 (7.9%) dog fecal samples from PS and in one of the 11 (9.1%) dog fecal samples from SM. Eggs of *Ancylostoma* spp. were detected in 27 (71.1%) dog fecal samples from PS, and in 7 (63.6%) dogs from SM. Other helminthes, like *Toxocara*, *Trichuris* and *Spirocerca*, were detected at a lower frequency (Table 1).

### Molecular detection of intestinal protozoa

The prevalence of protozoa in human samples as detected by PCR were as follows: in the PS village, 45% (40 of 88) for *Blastocystis* sp., 18.2% (16 of 88) for *G. duodenalis*, 13.6% (12 of 88) for *D. fragilis*, and 0% for *Cryptosporidium* spp., whereas in the SM village, 71% (27 of 38) for *Blastocystis* sp., 7.9% (3 of 38) for *G. duodenalis*, 18.4% (7 of 38) for *D. fragilis*, and 0% for *Cryptosporidium* spp. (Tables 2 and 3).

**Table 2:** **Genotypic characterization of** ***Blastocystis*** **,** ***Giardia duodenalis*** **and** ***Dientamoeba fragilis*** **isolates from humans in the Porto Said village (Tietê river, State of São Paulo, Brazil)**

**Sex**	**Age**	**Status**	***Blastocystis*** **subtype**	***G. duodenalis*** **assemblage**	***D. fragilis*** **genotype**
				***bg*** **/** ***tpi*** **/** ***gdh***	
F	45	Single	ST3	-	-
M	21	Single	ST3	-	-
M	21	Single	ST3	-	-
M	48	Single	-	-	-
F	43	Single	-	-	-
F	51	Single	-	-	-
M	48	Single	-	-/A1/-	-
M	23	Family 1	ST3	-	-
F	23	Family 1	-	-	1
M	6	Family 1	ST1	-	1
M	44	Family 2	ST1	-	-
F	43	Family 2	ST3	-	-
M	43	Family 3	-	-	-
F	58	Family 3	ST3	-	-
F	31	Family 3	ST1	-	1
F	12	Family 3	ST3	-	-
M	10	Family 3	ST1	-	1
M	4	Family 3	-	-	-
M	29	Family 4	ST3	-	-
M	40	Family 4	-	-	-
F	42	Family 5	ST3	-	1
M	43	Family 5	-	-	-
F	15	Family 5	-	-	1
M	7	Family 5	-	-	-
M	3	Family 5	-	-	-
M	45	Family 6	ST1	-	-
F	43	Family 6	-	-	-
M	10	Family 6	-	-	-
M	3	Family 6	-	-/-/A2	-
M	47	Family 7	ST1	-	-
F	26	Family 7	ST1	-	-
M	7	Family 7	ST1	-	-
F	4	Family 7	ST1	B/B/B	1
M	46	Family 8	-	-	-
F	46	Family 8	-	-	-
F	68	Family 9	ST1	-	-
F	47	Family 9	ST1	-	-
F	31	Family 9	-	-	-
M	14	Family 9	ST1	-	-
F	12	Family 9	-	-	-
M	10	Family 9	-	B/-/-	-
M	7	Family 9	ST1	-	-
F	5	Family 9	-	-	-
M	51	Family 10	ST1	-	1
F	38	Family 10	-	-	-
F	7	Family 10	-	A2/-/-	-
F	67	Family 11	-	-	-
M	59	Family 11	ST3	-	-
M	49	Family 11	ST3	-	-
M	44	Family 12	ST2	-	1
F	35	Family 12	ST1	-	1
F	15	Family 12	ST1	A2/-/-	-
M	13	Family 12	-	A2/A2/A2	-
M	10	Family 12	ST1	B/B/B	-
F	5	Family 12	-	B/B/B*	-
F	34	Family 13	-	-	-
F	13	Family 13	-	-	-
F	11	Family 13	ST3	-	-
M	31	Family 14	-	-	-
M	31	Family 14	ST3	-	-
F	51	Family 14	-	-	-
M	49	Family 15	-	-	-
F	42	Family 15	ST3	-	-
F	12	Family 15	-	-	-
M	59	Family 16	-	B/B/B*	-
F	69	Family 16	ST1	-	-
F	13	Family 16	-	-	-
F	9	Family 16	ST3	-	-
M	8	Family 16	ST2	A2/-/-	1
M	31	Family 17	ST1	-	-
F	26	Family 17	-	-	-
M	11	Family 17	-	B/B/B	-
M	3	Family 17	-	B/B/B	-
M	47	Family 18	-	-	-
F	28	Family 18	-	-	-
F	6	Family 18	ST1	-	-
M	42	Family 19	-	A2/A2/A2	-
F	36	Family 19	ST6	-	-
M	13	Family 19	-	A2/A2/-	-
F	5	Family 19	ST3	-	-
M	32	Family 20	-	-	-
F	26	Family 20	-	-	-
F	4	Family 20	-	-	-
M	31	Family 21	-	B	-
F	27	Family 21	-	-	-
M	3	Family 21	-	-	-
M	31	Family 22	-	-	-
F	39	Family 22	ST2	-	1

*gdh analysed by the method of Read et al. (2004) [17].

**Table 3:** **Genotypic characterization of** ***Blastocystis*** **,** ***Giardia duodenalis*** **and** ***Dientamoeba fragilis*** **isolates from humans in the Santa Maria village (Tietê river, State of São Paulo, Brazil)**

**Sex**	**Age**	**Status**	***Blastocystis*** **subtype**	***G. duodenalis*** **assemblage**	***D. fragilis*** **genotype**
				***bg tpi gdh***	
F	46	Single	ST3 + ST7	-	-
F	53	Single	ST3	-	-
F	36	Single	ST6	-/A2/-	-
M	42	Single	-	-	1
M	48	Single	ST1	-	-
F	43	Single	ST3	-	-
M	35	Single	ST3	-	-
M	75	Single	-	-	-
M	58	Single	-	-	-
F	62	Single	ST3	-	-
M	34	Single	ST1 + ST3	-	-
M	35	Single	-	-	-
M	25	Family 1	ST3	-	-
F	27	Family 1	ST1	-	-
M	2	Family 1	ST3	-	-
M	32	Family 2	ST2	-	-
F	27	Family 2	ST7	-	-
M	6	Family 2	ST3	-	1
M	7	Family 3	ST3	-	1
F	36	Family 3	-	-	-
M	35	Family 3	ST7	-	-
F	10	Family 3	ST3	-	-
F	12	Family 3	-	B/B/B	1
F	41	Family 4	ST1	A2/-/-	1
M	9	Family 4	-	-	-
M	55	Family 5	-	-	-
F	49	Family 5	ST7	-	-
F	4	Family 6	ST2	-	-
F	24	Family 6	ST3	-	-
M	4	Family 7	-	-	-
F	62	Family 7	ST7	-	-
F	6	Family 7	ST3	-	1
M	32	Family 7	ST2	-	-
F	4	Family 7	-	-	-
F	26	Family 7	ST3	-	-
M	64	Family 8	ST2	-	1
F	47	Family 8	ST3	-	-
M	37	Family 8	-	-	-

### Analysis of single and multiple infections

In individuals from PS, double infections with *Blastocystis* sp. and *G. duodenalis* (n = 2; Table 2) were found to occur at a frequency lower than expected (Chi-square test, p = 0.011), whereas double infections with *Blastocystis* sp. and *D. fragilis* (n = 8; Table 2) were found at a frequency higher than expected (Chi-square test, p = 0.019). Two cases of triple infections were observed. As expected, the prevalence of *G. duodenalis* infection was higher in children compared to adults (data not shown), but this does not explain the low number of co-infection with *Blastocystis* sp., as this parasite was also commonly detected among children.

In individuals from SM, double infections with *Blastocystis* sp. and *G. duodenalis* or with *Blastocystis* sp. and *D. fragilis* occurred at the expected frequency. One case of triple infection was observed (Table 3).

When the data from both villages were combined, double infections with *Blastocystis* sp. and *G. duodenalis* (n = 3) were found to occur at a frequency lower than expected (Chi-square test, p = 0.003), whereas double infections with *Blastocystis* sp. and *D. fragilis* (n = 12) were found at a frequency higher than expected (Chi-square test, p = 0.057).

We also investigated the distribution of *Blastocystis* STs in PS (Table 2) and noted that 15 of 16 cases of infection with ST3 occurred as single infections compared to 13 of 20 for ST1 (Fisher’s test, p = 0.002). However, this was not observed in SM (Table 3), where ST3 was found in combination with *D. fragilis* (3 cases) or with other *Blastocystis* STs (one with ST7 and one case with ST1).

### Typing of *Blastocystis* sp.

Among individuals from PS, sequencing of a fragment of the ssu rDNA gene indicated the presence of subtypes ST1 (20 samples), ST2 (3 samples), ST3 (16 samples) and ST6 (1 sample). BLAST searches at the *Blastocystis* subtype database further allowed us to identify alleles 4 and 81 within ST1, alleles 11 and 12 within ST2, alleles 34, 36 and 37 within ST3 (Table 2). Mixed infections with two ST1 alleles (4 and 81), two ST3 alleles (34 and 36), and two ST6 alleles (122 and 134) were each found in single samples (Table 2).

Among individuals from SM, sequencing indicated the presence of ST1 (3 samples), ST2 (4 samples), ST3 (13 samples), ST6 (1 sample) and ST7 (4 samples) subtypes. BLAST search at the *Blastocystis* allele database further allowed to identify allele 4 of ST1, alleles 9, 10 and 12 of ST2, alleles 34, 36 and 37 of ST3, and allele 96 of ST7 (Table 3). Mixed infections with two ST3 alleles (36 and 128), two ST6 alleles (122 and 134) and two ST7 alleles (96 and a novel allele) were each found in single samples. Two mixed infections with different subtypes (one with ST3 + ST7, and one with ST1 + ST3) were found after cloning of the PCR products and sequencing of five individual plasmids (Table 3). Phylogenetic analysis confirmed that the *Blastocystis* samples belonged to strongly supported clusters, i.e., to different STs (data not shown).

All dog samples (38 from PS and 11 from SM) were negative for *Blastocystis* by PCR.

### Typing of *Giardia duodenalis*

Among individuals from PS, sequencing revealed assemblage A in 8 samples and assemblage B in 9 samples (Table 2). Among assemblage A samples, genotype A1 was found at the *tpi* locus in 1 isolate, whereas genotype A2 was found in all other samples, regardless of the marker (Table 2).

Among assemblage B samples, a single genotype was found at the *tpi* locus, which was identical to that reported in different hosts (humans and a dog) and in a wastewater sample. At the *bg* locus, 3 different genotypes were found: the first was identified in 5 samples, and had 100% identity with previously described samples from humans and a macaque; the second was found in 2 samples, had 100% identity with previously described samples from humans, from a calf and from reference strains; the third was found in 1 isolate, had 100% identity with a previously described human isolate from Uganda. At the *gdh* locus, two genotypes were found: the first was identified in 4 samples and had 100% identity with previously described samples from humans, whereas the second, found in 1 isolate, had 100% identity with animal samples (chinchillas and a rabbit) and with reference strains (Ad-45 and Ad-28). Two samples were analyzed by sequencing a different *gdh* fragment (Table 2), and two genotypes were found, one having 100% identity with a single human isolate, whereas the other had 99% identity with many samples, including the reference strain Ad-45 and Ad-28.

Among humans from SM, assemblage A was found in 2 samples, and genotype A2 was identified at the *bg* or *tpi* locus, respectively (Table 3). A single isolate was typed as assemblage B at 3 loci, and the same genotypes found in the PS village were identified.

Regardless of the village and the *G. duodenalis* assemblage, inspection of sequencing files did not show the presence of double peaks (overlapping nucleotides at specific positions), indicating a lack of allelic sequence heterogeneity in these samples. For 7 samples from assemblage B, sequence information was available at 3 loci (Tables 2 and 3), and two multi-locus genotypes (MLG) were detected, one found in 5 samples and the other in 2 samples. Phylogenetic analyses of single or concatenated genes showed a closer relationship of Brazilian samples with reference sequences of assemblage B, sub-assemblage BIV (data not shown).

A total of 49 dog fecal samples were tested by PCR, and amplification of at least one gene fragment was obtained from 6 samples, 4 from PS and 2 from SM. Genotype A2 was identified in 2 dogs from PS (at the *bg* and *tpi* loci, and at the *bg* and *gdh* loci, respectively) and in one dog from SM (at the *bg* locus). Assemblage C was identified at the *tpi* locus in one dog from PS, and at all the 3 loci in one dog from SM. Assemblage D was identified at the *bg* and *gdh* loci in one dog from PS.

### Typing of *Dientamoeba fragilis*

Sequencing of the PCR products from the 19 human samples positive for *D. fragilis* (12 from PS and 7 from SM) showed the presence of genotype 1 in all samples (100% identity to AY730405; Tables 2 and 3).

All dog samples (38 from PS and 11 from SM) tested negative by PCR.

### Typing of *Cryptosporidium* spp.

Sequencing of a fragment of the SSU rDNA gene from the single dog sample positive revealed the presence of *Cryptosporidium canis* (100% identity to GenBankAB210854).

### Analyses of river water samples

The analysis of 28 river water samples did not reveal the presence of *Cryptosporidium* oocysts or *Giardia* cysts, based on results of both microcopy and PCR. The levels of turbidity of water samples collected during the dry (average values, PS = 8.66 NTU, SM = 12.64 NTU) and the rainy (average values, PS = 28.53 NTU, SM = 20.59 NTU) seasons, as well as the pH values (close to neutrality) were within a range which is considered appropriate for the applied methodology.

The DNAs extracted from water samples were also tested by PCR for *Blastocystis* sp., and amplification was observed from 3 samples collected in PS. Direct sequencing of PCR products generated overlapping chromatograms, likely arising from amplification of more than one DNA template (data not shown). The corresponding PCR products were cloned and individual plasmids (n = 12) were sequenced. This allowed the identification of sequences having significant identity (99%) to an uncultured stramenopile identified in a study of the lake Taihu, China (GenBank GQ844623), and to an uncultured dictyochophyte identified in a study of the lake Eschsur Sure, Luxembourg (GenBankGU068032). These eukaryotic microorganisms were both present in the 3 water samples from PS, whereas *Blastocystis* sp. was not identified in any of these samples.

## Discussion

The main objective of this study was to investigate the occurrence and the genetic diversity of intestinal protozoa in the stools of asymptomatic individuals living in two villages located along the Tietê river, State of São Paulo, Brazil. To gain insights about possible transmission routes, fecal samples from roaming dogs and water samples from the river were also collected. All samples were tested by microscopy and PCR. As expected, PCR detected a higher number of human infections than microscopy, likely because of the small number of parasites shed with the feces of asymptomatic individuals, or, in the case of *D. fragilis* and *Cryptosporidium* spp., because microscopic detection requires permanent staining procedures which were not used in this study.

We will first discuss some general implications of the present findings. As mentioned above, overt intestinal symptoms were not observed among the individuals from the two villages, yet they were frequently infected with one or more parasites. A comparison of the prevalence of different protozoa in the two villages showed that double infections with *Blastocystis* sp. and *G. duodenalis* occurred at a frequency lower than expected in individuals from the PS village, and that this result was not explained by the higher frequency of *G. duodenalis* among young individuals. The same trend was not observed in the SM village, albeit it should be noted that *Blastocystis* sp. was highly prevalent in this village while *G. duodenalis* was rarely found. We also observed that double infections with *Blastocystis* sp. and *D. fragilis* occurred at a frequency higher than expected in individuals from the PS village, but not in those from the SM village. An association between *Blastocystis* sp. and *D. fragilis* has been recently reported in a study of children presenting with gastrointestinal symptoms [20]. Furthermore, we noticed that single infections with *Blastocystis* ST3, but not with ST1, were found at a frequency higher than expected in individuals from the PS village; however, this was not observed in those from the SM village. Further investigations are needed to understand whether these observations reflect real biological interactions among different protozoa that colonize the human gut.

Other surveys conducted in Brazil, both in rural and urban contexts [5-10], also identified frequent asymptomatic infection caused by *Blastocystis* sp. and *G. duodenalis*. This suggests that, where infections are endemic, humans elicit an immune reaction that minimizes the deleterious effects of a given parasite (immunological tolerance) without achieving parasite clearance, a concept well established in the case of helminthic infections [21]. On the other hand, there is clear evidence that *Cryptosporidium* and *Giardia* can cause severe diarrhea in children, particularly in malnourished children, with a significant impact on growth and cognitive function [22]. Thus, understanding which parasite and host factors are responsible for acute enteritis and long term adverse health effects remains an important area of research.

Some interesting comments were derived from the molecular characterization of intestinal protozoa that was performed. In the case of *Blastocystis* sp., which was the most common human parasite in both villages, the most remarkable finding was the absence of ST4, which confirmed previous studies from South America [23-25]. Indeed, ST4 has been identified only in a few non-human primates in South America [25,26], in contrast with data from Europe where ST4 has been linked to symptomatic infection and to irritable bowel syndrome in humans [26]. Further studies are needed to understand the origin of this ST, which, compared to other human-associated STs, displays lower genetic variability, thus suggesting its recent acquisition as a parasite of humans [27]. Moreover, when the PCR assay that is commonly used to detect *Blastocystis* sp. in feces [13] was applied to water samples, other stramenopiles were identified. In particular, sequences with very high homology (99%) to stramenopiles from freshwater lakes were identified in three water samples from the PS village. As commented in the original article [13], the primers were developed to detect *Blastocystis* sp. in fecal samples from vertebrate hosts or from *in vitro* cultures, and not from environmental samples. The investigators noticed the homology of the primers to other stramenopiles, but considered it not to be relevant. To our knowledge, only a few studies have applied PCR to detect and type *Blastocystis* sp. in water samples. In a study from Turkey, tap water samples were tested using subtype-specific sequenced-tagged site primers, and ST1 was identified in 3 of 25 samples [28]. In central Nepal, water samples from two rivers were tested using subtype-specific sequenced-tagged site primers and found to contain *Blastocystis* ST1 and ST6 [29]. In waste water samples assessed in the Philippines [30], *Blastocystis* ST1 and ST2 subtypes were identified by PCR of full length ssu rDNA [31], but detection was done after *in vitro* cultivation of the parasite. Our findings highlight a need to test existing primers, and eventually design new ones, for the specific amplification of *Blastocystis* sp. from water samples, and even from food samples.

In the case of *G. duodenalis*, typing of human samples showed the presence of sub-assemblage AII (mainly genotype A2) and sub-assemblage BIV. A literature survey of other studies on human giardiasis in Brazil showed that genotype A1 (sub-assemblage AI) was identified more often (98 samples) than genotype A2 (sub-assemblage AII, 47 samples) [32,33]. This contrasts with the data obtained in Europe and other parts of the world where genotypes of sub-assemblage AII (mainly genotype A2) predominate in humans [34]. On the other hand, the identification of the BIV sub-assemblage is in agreement with a previous study of 8 human samples from Brazil based only on *gdh* sequencing [33]. Typing of *G. duodenalis* samples from dogs revealed both zoonotic (A2) and non-zoonotic(C and D) assemblages. The occurrence of genotype A2 likely reflects the exposure of dogs to cysts of human origin, resulting from an inadequate disposal of sewage in the PS and SM villages. In previous studies from Brazil, both zoonotic (genotypes A1 and A2) and non-zoonotic (assemblages C and D) strains were detected in dogs [32,33]. The role of dogs as reservoirs of zoonotic assemblages of *G. duodenalis* has been widely discussed in the literature [35-37]. The available data show that the host-specific assemblages are largely more prevalent than zoonotic assemblages, worldwide, and only a few surveys conducted in poor settings have provided evidence for a transmission cycle between humans and dogs [38].

In the case of *D. fragilis*, this is the first study to characterize samples from South America at the molecular level. Sequencing of an ssu rDNA gene fragment revealed the presence of genotype 1 in all of the 19 positive human samples. Information about this parasite is scanty, but of the 2 genotypes (1 and 2) described until now [39], genotype 1 is by far the most prevalent, and our data confirm this observation.

Finally, the absence of *Cryptosporidium* spp. was not surprising, as infection with this parasite is usually symptomatic and associated with watery diarrhea, abdominal pain, nausea and vomiting. A symptomatic carriage has been reported, but detection of the very low number of oocysts in the feces remains challenging [40]. The absence of *Cryptosporidium* oocysts in the river water samples tested, and the finding of *Cryptosporidium canis* in the single positive dog, suggest a limited circulation of *Cryptosporidium* species pathogenic to humans in the study area.

This study has some limitations. First, only one staining technique was used for the microscopic detection of parasites and stool samples were preserved in potassium dichromate; this may have affected the detection of some species, particularly *D. fragilis* and *Cryptosporidium* spp. Second, we have been unable to collect fecal samples from chickens and cats, and thus to investigate their potential role in the transmission of intestinal protozoa to humans. It is noticeable that in the SM village, infection with *Blastocystis* ST6 and ST7 have been detected, and, since birds are the main host of these STs, it would have been valuable to test chickens for the presence of this parasite. More generally, strong conclusions about the routes of transmission in the two villages could not be reached. The fact that different *Blastocystis* STs and different *G. duodenalis* assemblages were found among members of a single family indicates multiple sources of exposure and suggests a role of the environment in the transmission. On the other hand, the detection of a single ST or assemblage in other families is more likely explained by direct person-to-person transmission within households.

## Conclusions

A high carriage of intestinal protozoa characterized the populations under study, and many individuals carried multiple parasites in the absence of apparent gastrointestinal symptoms. The large genetic heterogeneity observed among samples of *Blastocystis* and *Giardia* suggests exposure to multiple sources, including person-to-person contacts and, possibly, contaminated water or food.
